# Identification of ferroptosis related biomarkers and immune infiltration in Parkinson’s disease by integrated bioinformatic analysis

**DOI:** 10.1186/s12920-023-01481-3

**Published:** 2023-03-14

**Authors:** Na Xing, Ziye Dong, Qiaoli Wu, Yufeng Zhang, Pengcheng Kan, Yuan Han, Xiuli Cheng, Yaru Wang, Biao Zhang

**Affiliations:** 1 Clinical College of Neurology, Neurosurgery and Neurorehabilitation, Tianjin Medical University, Tianjin, China; 2 Tianjin Key Laboratory of Cerebral Vascular and Neurodegenerative Diseases, Tianjin Neurosurgical Institute, Tianjin Huanhu Hospital, Tianjin, China; 3 Department of Clinical Laboratory, Tianjin Huanhu Hospital, Tianjin, China; 4 Chu Hsien-I Memorial Hospital (Metabolic Diseases Hospital) of Tianjin Medical University, Tianjin, China

**Keywords:** Parkinson’s disease, Ferroptosis, Immune infiltration, Immune checkpoint gene, ELISA, Bioinformatic

## Abstract

**Background:**

Increasing evidence has indicated that ferroptosis engages in the progression of Parkinson’s disease (PD). This study aimed to explore the role of ferroptosis-related genes (FRGs), immune infiltration and immune checkpoint genes (ICGs) in the pathogenesis and development of PD.

**Methods:**

The microarray data of PD patients and healthy controls (HC) from the Gene Expression Omnibus (GEO) database was downloaded. Weighted gene co-expression network analysis (WGCNA) was processed to identify the significant modules related to PD in the GSE18838 dataset. Machine learning algorithms were used to screen the candidate biomarkers based on the intersect between WGCNA, FRGs and differentially expressed genes. Enrichment analysis of GSVA, GSEA, GO, KEGG, and immune infiltration, group comparison of ICGs were also performed. Next, candidate biomarkers were validated in clinical samples by ELISA and receiver operating characteristic curve (ROC) was used to assess diagnose ability.

**Results:**

In this study, FRGs had correlations with ICGs, immune infiltration. Then, plasma levels of LPIN1 in PD was significantly lower than that in healthy controls, while the expression of TNFAIP3 was higher in PD in comparison with HC. ROC curves showed that the area under curve (AUC) of the LPIN1 and TNFAIP3 combination was 0.833 (95% CI: 0.750–0.916). Moreover, each biomarker alone could discriminate the PD from HC (LPIN1: AUC = 0.754, 95% CI: 0.659–0.849; TNFAIP3: AUC = 0.754, 95% CI: 0.660–0.849). For detection of early PD from HC, the model of combination maintained diagnostic accuracy with an AUC of 0.831 (95% CI: 0.734–0.927), LPIN1 also performed well in distinguishing the early PD from HC (AUC = 0.817, 95% CI: 0.717–0.917). However, the diagnostic efficacy was relatively poor in distinguishing the early from middle-advanced PD patients.

**Conclusion:**

The combination model composed of LPIN1 and TNFAIP3, and each biomarker may serve as an efficient tool for distinguishing PD from HC.

**Supplementary Information:**

The online version contains supplementary material available at 10.1186/s12920-023-01481-3.

## Background

Parkinson’s disease (PD) is a common neurodegenerative disorder which involves in classic motor features of Parkinsonism including tremor, akinesia and bradykinesia, as well as nonmotor symptoms such as constipation, sleep disturbance and cognitive impairment and so on [1, 2]. The typical pathologic characteristics of PD are pathologic accumulation of cytoplasmic misfolded α-synuclein, in form of Lewy bodies and progressive loss of dopaminergic neurons in the substantia nigra pars compacta (SNpc). The incidence increases with age while relatively little is known of the exact neurodegenerative pathogenesis, which relates to multiple factors including genetics, oxidative stress, immune activation, mitochondrial dysfunction or lipid dyshomeostasis [3].

Ferroptosis, an iron-dependent non-apoptotic regulated programmed cell death, was firstly proposed in 2012 [4], which is mainly driven by iron dyshomeostasis and lipid peroxidation, leading to oxidative stress in cells and affecting metabolic processes of protein, nucleic acid, carbohydrates and lipids, ultimately leads to cell death [4, 5]. However, ferroptosis distinguishes from apoptosis, necrosis, autophagy and other forms of cell death in morphologically, biochemically and genetically [4]. Previous studies on ferroptosis mainly focused on cancer, and iron metabolism has become a hot spot in tumorigenesis, progression and treatment prognosis. To data, ferroptosis-related genes (FRGs) have been recognized as diagnostic biomarkers for multiple cancers [6, 7]. A rat organotypic hippocampal slice culture model showed that erastin induced ferroptosis can promote neuronal death by creating a void in the antioxidant defenses of cell, but Fer-1 prevents glutamate-induced neurotoxicity [4]. Iron is an oxidant and excess free iron can induce oxidative stress, inflammation and excitotoxicity, causing cellular damage and neurodegeneration [8, 9]. The dyshomeostasis and intracellular retention of iron are associated with senescence of mutiple types of cells, including neurons, which accelerates aging by inducing DNA damage and blocking genomic repair systems [10].

A recent discovery that α-synuclein oligomers can bind to the plasma membrane and drive cell ferroptosis via altered membrane conductance, abnormal calcium influx and lipid peroxides production, which provides the direct evidence that ferroptosis is referred as an essential pathogenic mechanism in synucleinopathies [11]. Ever increasing evidence linking α-synuclein to the metabolism of iron and lipids, suggesting a possible role of α-synuclein in ferroptosis [11]. Previous research has found that selective iron deposition pattern in substantia nigra is greatly influenced by the age of PD onset [12]. Activated glia promote dysregulation of iron homeostasis, thereby aggravating microglial activation, which plays a pivotal role in ferroptosis and subsequent neurodegeneration [13]. Characteristics of ferroptosis, such as iron accumulation, glutathione (GSH) depletion, lipid peroxidation and elevated reactive oxygen species (ROS), may be observed in PD patients [14]. Moreover, ferric ammonium citrate (FAC)-induced ferroptosis in dopaminergic cells is related to the phosphorylation of p53 signaling pathway not MAPK pathway [15]. However, the controversial results in erastin-treated Lund human mesencephalic cells indicate that whether erastin-induced ferroptosis is RAS-dependent needs further investigation [16, 17]. Conservative iron chelation modality (avoiding changes in systemic iron levels) established in mammalian models and clinical trials that offers a new therapeutic strategy based on iron scavenging and redeployment for neuroprotection [18].

In the present study, we investigated the biological pathways of pathophysiology from the perspective of ferroptosis in PD based on bioinformatics analysis and identified gene co-expression modules by WGCNA, further examine the relationship of FRGs with immune infiltration and immune checkpoint genes (ICGs). Moreover, the expression profiles of candidate genes were detected in clinical blood samples. The possible role and function of core genes in regulating ferroptosis and immune infiltration in PD were also explored.

## Methods

**Fig. 1:**
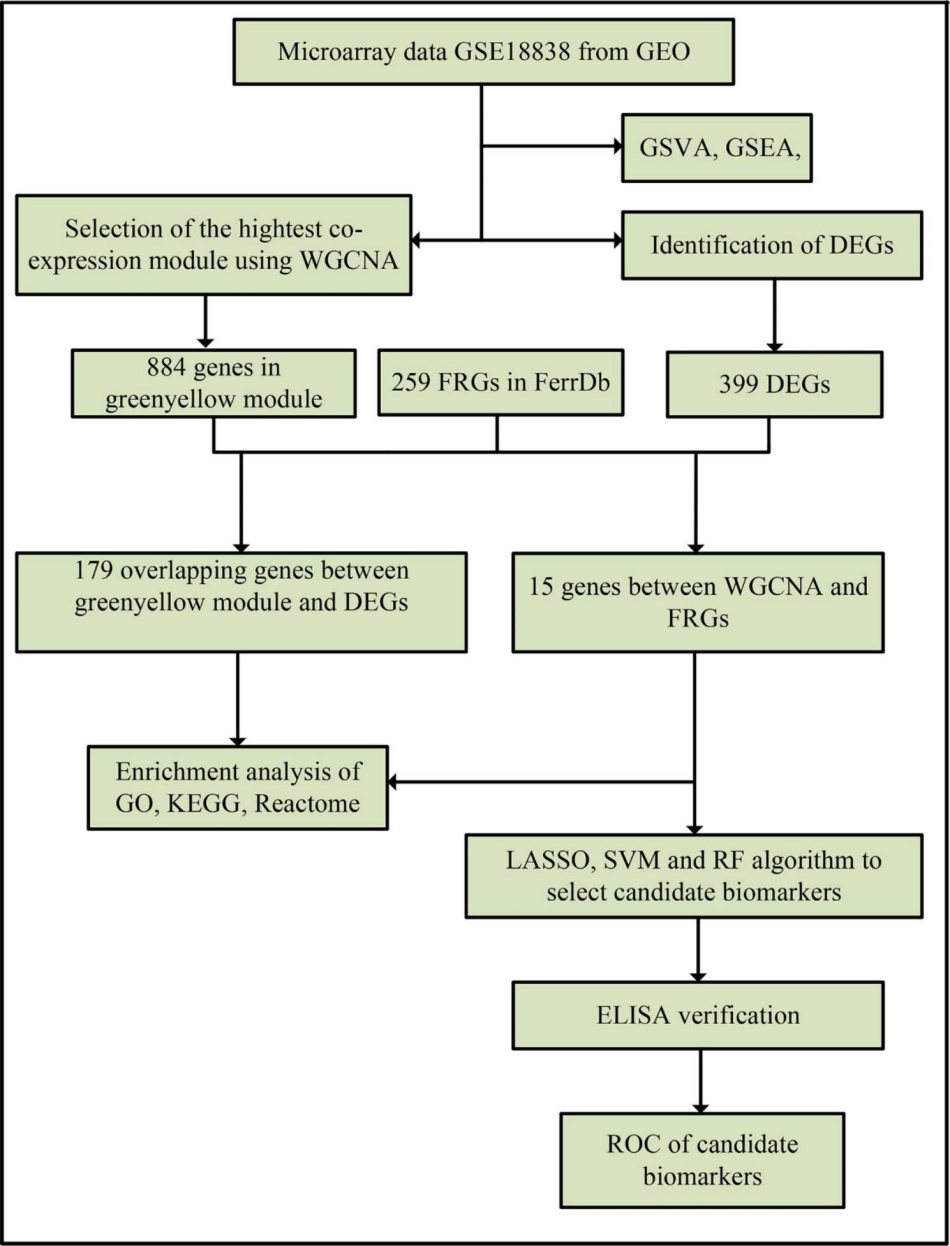
The work flow of this study

The work flow of this study is shown in Fig. 1.

### Data acquisition and preprocessing

We applied “Parkinson’s disease”, “human beings”, “peripheral blood”, “expression profiling by array” as key words and ensured that each group has more than 10 subjects, the gene expression matrix of GSE18838 dataset [19] was obtained from the NCBI Gene Expression Omnibus (GEO) database (https://www.ncbi.nlm.nih.gov/geo/) (Accessed: 1 May 2022). The GSE18838 dataset included 17 PD and 11 healthy control (HC) whole blood samples, which was performed on GPL5175 platform ([HuEx-1_0-st] Affymetrix Human Exon 1.0 ST Array [transcript (gene) version]). The clinical characteristics of participates in GSE18838 are detailed in (Additional file 1: Table S1).

FerrDb (http://www.zhounan.org/ferrdb/) (Accessed: 2 May 2022) collected 259 ferroptosis-related genes (FRGs) including driver, suppressor and marker [20]. The confidence level of recorded genes involved in ferroptosis was assigned to 4 degrees including validated, screened, predicted and deduced.

### WGCNA analysis and intersect between DEGs and interesting module

In this study, we utilized a gene expression profile of GSE18838 to construct a weighted gene co-expression network between PD and HC using the “WGCNA” package in R software [21] and analyzed the relationships between gene modules and clinical phenotype of PD. Briefly, cluster analysis was used to explore whether there was outlier samples in the GSE18838 dataset to ensure the accuracy of further exploration. According to the scale-free topology criterion, we used the function pickSoftThreshold to select soft powers β = 12 and the soft thresholding parameter showed strong relations between genes while penalized the weak correlation. Then, the adjacency matrix was transformed into a topological overlap matrix (TOM) to measure the network connectivity of genes as well as the corresponding dissimilarity (1-TOM). A hierarchical clustering tree diagram of the 1-TOM matrix was constructed to classify genes showing similar expression profiles with gene co-expression modules. Then dynamic tree cut method was performed to separate different modules of all genes and merged the similar models using MEDissThres = 0.25. The different branches represented a different module. Subsequently, module-trait relationships were estimated via pearson analysis and the module with high correlation coefficients was considered as interesting module. The genes in the module were selected for following research.

Differentially expressed genes (DEGs) between PD and HC were identified utilizing the “limma” package in R software based on the following threshold: p-value < 0.05 and |log2FC|>0.5 in the GSE18838 dataset. The p-value was adjusted by Benjamini–Hochberg method to control the false discovery rate (FDR). The DEGs were visualized as volcano plot by using " ggplot2” package in R software.

Then, the intersect between DEGs, FRGs and co-expression genes that were extracted from interesting module was visualized as Venn diagram.

### Enrichment analysis of GSVA and GSEA

Gene set variation analysis (GSVA) was performed on the expression profile of GSE18838 using “GSVA” package in R software and the reference gene sets were hallmark gene sets, GO-BP, GO-CC, GO-MF, KEGG and C7: immunologic signatures, which were downloaded from the MSigDB database (https://www.gsea-msigdb.org/gsea/msigdb) [22] (Accessed: 2 May 2022). Gene Set Enrichment Analysis (GSEA) was operated using “GSEA” R package to investigate relate pathways of the candidate diagnostic genes and the reference gene set were KEGG. The number of random sample permutations was set at 1000, p < 0.05 was considered as significant enrichment.

### Machine learning algorithm for candidate genes

After identifying DEGs, we performed three machine learning algorithms as least absolute shrinkage and selection operator (LASSO) logistic regression, random forest (RF) and support vector machine-recursive feature elimination (SVM-RFE) to screen candidate genes for PD using “glmnet”, “randomforest”, and “e1071” package in R software, respectively. Then, we combined the genes from LASSO, RF and SVM-RFE algorithms for further analysis. The expression of the candidate gene was firstly validated in GSE18838 dataset and a two-sided p < 0.05 was considered statistically significant. Ultimately, the area under the receiver operating characteristic (ROC) curve analysis (AUC) was calculated to evaluate the accuracy of selected genes for diagnosing PD patients. The transcription factor (TF)-miRNA coregulatory network was constructed on NetworkAnalyst (https://www.networkanalyst.ca) (Accessed: 2 June 2022).

### GO and KEGG analysis

To explore the potential molecular mechanism of key genes associated with PD, Gene Ontology (GO) including biological process (BP), cellular component (CC) and molecular function (MF), and Kyoto Encyclopedia of Genes and Genomes (KEGG) analyses were operated using “clusterProfiler” R package [23]. The metascape database (http://metascape.org/) (Accessed: 10 June 2022) is an online database used for gene annotation, functional enrichment, interactome and membership analysis, used for KEGG and Reactome pathway analysis in the present study. p value < 0.05 as the screening threshold.

### Infiltration of immune cells and correlation analysis

The “CIBERSORT” algorithm was applied to calculate the ratios of immune infiltrating cells in PD and HC samples [24]. The number of permutations of default signature matrix was set to 1000 and the standard immune cell expression file (LM22.txt) was obtained from official website (https://cibersort.stanford.edu/) (Accessed: 7 June 2022). The different proportion of immune cells and expression of immune checkpoint genes associated with T cells (Additional file 2: Table S2) between two groups were detected by Wilcoxon rank sum test [25] and spearman correlation analysis was performed on candidate genes and infiltrating immune cells, ICGs.

### Patient enrollment and blood acquisition

70 PD patients and 39 healthy controls were recruited in this study at the Tianjin Huanhu Hospital. The Ethics Committee of Huanhu Hospital approved this study and written informed consent was obtained from all study participants. Disease severity was evaluated using the modified Hoehn and Yahr (H&Y) scale. PD patients were divided into early stage (early) and middle-advanced stages (mid-advanced) groups according to their HY scale. Early stage contained 30 patients (H&Y sca1e 1-2.5) and middle-advanced stage included 40 patients (H&Y scale 3–5). The scale of MDS-UPDRS III (MDS Unified-Parkinson Disease Rating Scale) was used to examine movement function of PD patients. All patients were diagnosed by at least 3 professional and fellowship-trained movement disorders neurologists according to the UK Society Brain Bank Criteria for the diagnosis of PD. Healthy control subjects had no personal or family history of neurodegenerative diseases. Exclusion criteria were as follows: a history of deep brain stimulation and anticancer therapy; major depression; dementia; hepatorenal disease; stroke or other cerebrovascular disease.

2ml EDTA-K2 anticoagulant whole blood was collected in the morning after the subjects fasted for 10 h. Blood was centrifuged at 1000 g for 15 min at room temperature to obtain plasma then stored at -80℃ for further analysis.

### Enzyme-linked immunosorbent assay

Plasmic concentrations of LPIN1 and TNFAIP3 in PD and HC were determined by commercially available enzyme-linked immunosorbent assay (ELISA) kits obtained from Herbal Source (Nanjing, China) and CUSABIO (Wuhan, China), respectively. The assay was performed according to the manufacturer’s instructions and the results were detected using SpectraMax iD5 multifunctional microplate reader at 450 nm (Molecular Devices, the USA).

### Statistical analysis

All data were analyzed using SPSS statistical software (version 26.0), GraphPad Prism software (Version 8.0) and R software (version 4.3.1; including “GEOquery”, “limma”, “WGCNA”, “FactoMineR”, “clusterProfiler”, “GSVA”, “GSEA”, “glmnet”, “randomforest”, “e1071”, “CIBERSORT”, “pROC”, “ggplot2” and “stats” package). For all analysis, p value < 0.05 was considered statistically significant. Data normality was first evaluated using Shapiro–Wilk test, then t test was used to compare data with normal distribution between two groups, and Mann-Whitney U test was used to compare data of non-normal distribution between two groups. One-way ANOVA analysis or Kruskal-Wallis test was used to compare data among three groups. Data were presented as mean ± standard deviation (SD) or median (quantile). Chi-square test was used for comparing sex ratios between PD patients and healthy controls. Receiver operating characteristic (ROC) curves were generated to evaluate their sensitivities and specificities in distinguishing PD from the healthy controls.

## Results

### Identification of key WGCNA module and DEGs

**Fig. 2:**
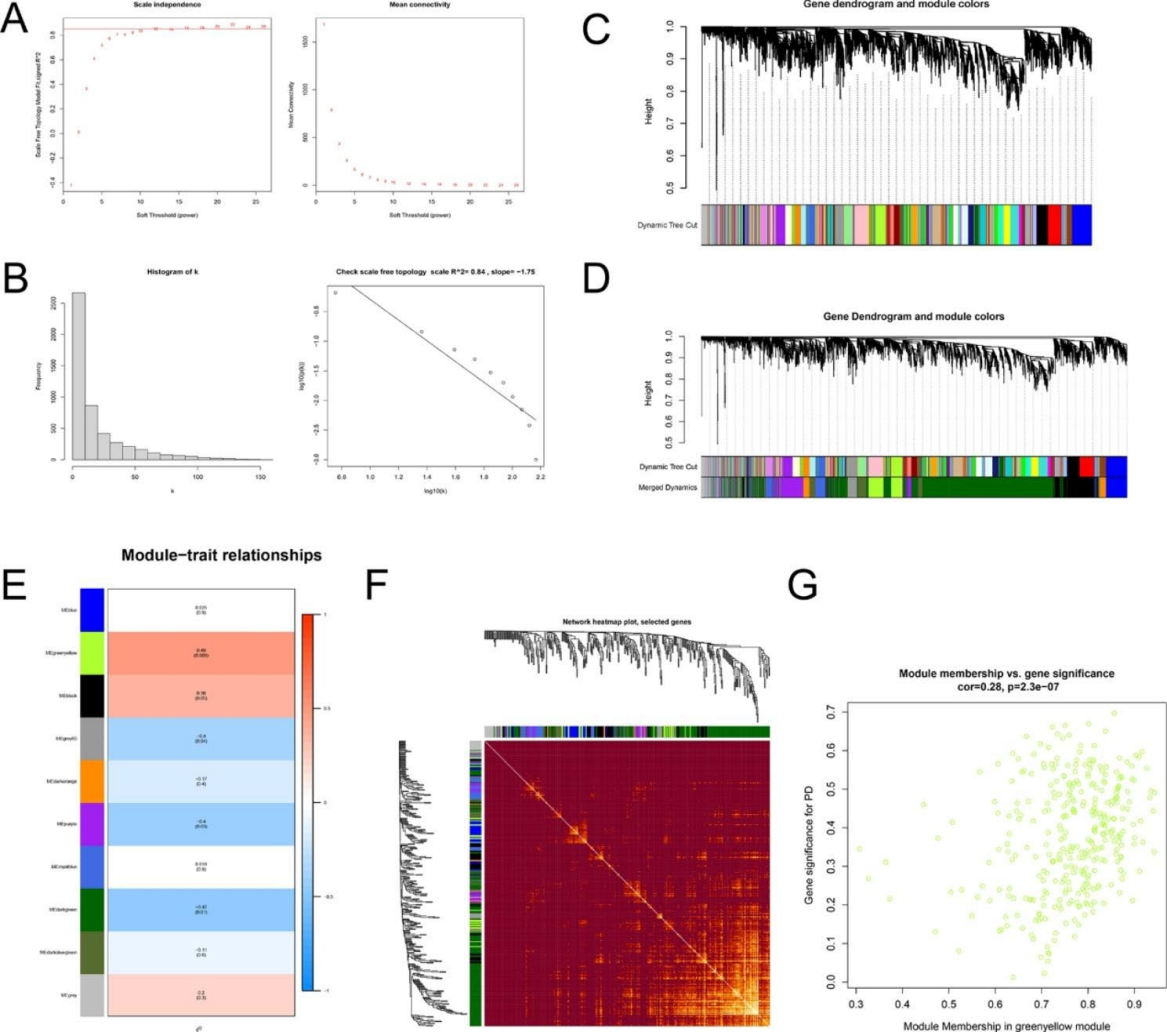
WGCNA network and module detection. **A** Selection of the soft-thresholding powers. The left panel displays the scale-free index versus soft-thresholding power. The right panel shows the mean connectivity versus soft-thresholding power. The x-axis represents weighting parameters (power). The y-axis represents the scale-free fit index and connectivity for each power. **B** Histogram of the number of node connections and validation that the network conforms to a scale-free distribution at a given threshold. **C** Module division. **D** Module merge. Each color represents a module in the co-expression network by WGCNA. **E** Heatmap of the correlation between module and PD samples traits. **F** The heatmap visualizing the gene network. **G** Scatterplot showing the correlation between gene significance and module membership in the greenyellow module

After the cluster analysis, no samples were removed (Additional file 3: Figure S3). The WGCNA network was constructed based on the GSE18838 dataset to identify the meaningful gene modules related with PD. A soft threshold power of 12 was selected, the scale-free topology fit index R^2 reached 0.84, and mean connectivity is 18.10, indicating that a scale-free network was established (Fig. 2A, B). Co-expression gene modules were identified through the dynamic tree cut method, after merging similar modules, the key modules were further screened based on MEDissThres = 0.25 (Fig. 2C, D). Then we analyzed the relationship between the key modules and clinical phenotype, and the heatmap of all genes in the key modules was displayed (Fig. 2E, F). Among the 10 modules analyzed, the greenyellow module was significantly associated with the clinical traits of PD and was chosen as a key module (cor = 0.49, p = 0.008, Fig. 2E). We selected 884 genes for following research according to the criterion of q.weighted < 0.05. Besides, a high correlation was observed between PD and the greenyellow module (cor = 0.492) while the correlation between module memberships (MM) and gene significance (GS) in the greenyellow module is 0.28 (cor = 0.28, p = 2.3e-07, Fig. 2G).

**Fig. 3:**
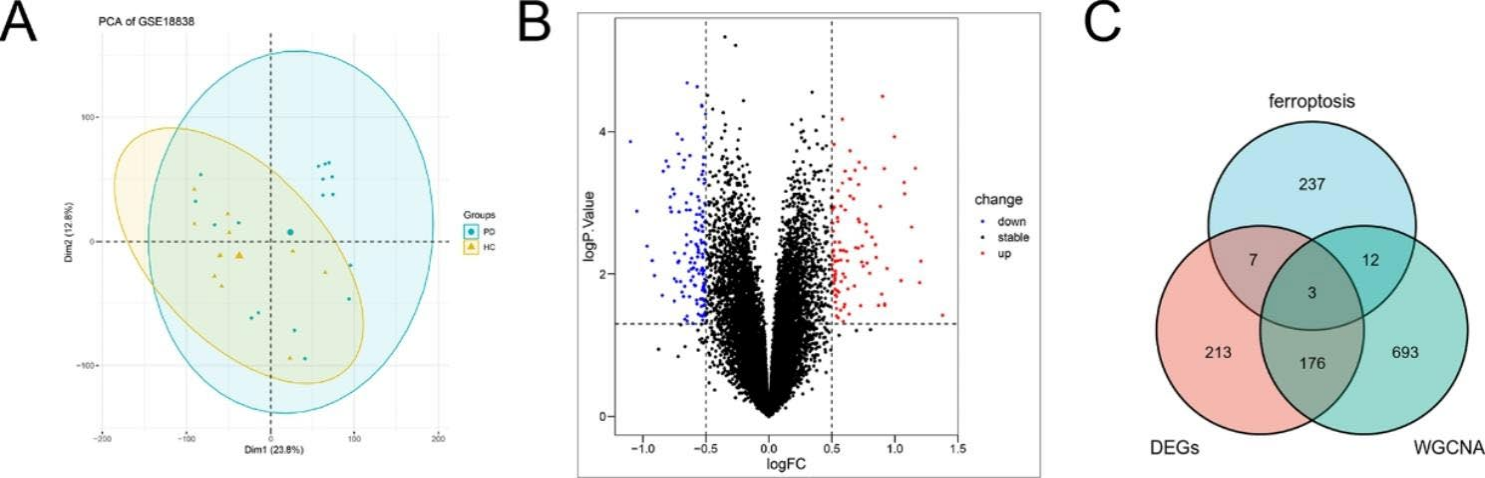
PCA plot of gene chip and volcano plot of different expression genes. **A** PCA analysis plot of GSE18838 gene chip. **B** Volcano plot of differential expressed genes between PD and HC samples in GSE18838 dataset. **C** Venn diagram displaying the overlap between DEGs, FRGs and PD-related genes identified by WGCNA.

Additionally, 399 DEGs between PD and HC samples were obtained through the PCA and different expression analysis (Fig. 3A, B). The intersect between DEGs, FRGs and co-expression genes in interesting module was visualized by Venn plot (Fig. 3C), thus we screened 15 ferroptosis-related-WGCNA genes and 179 WGCNA-DEGs.

### GSEA and GSVA

**Fig. 4:**
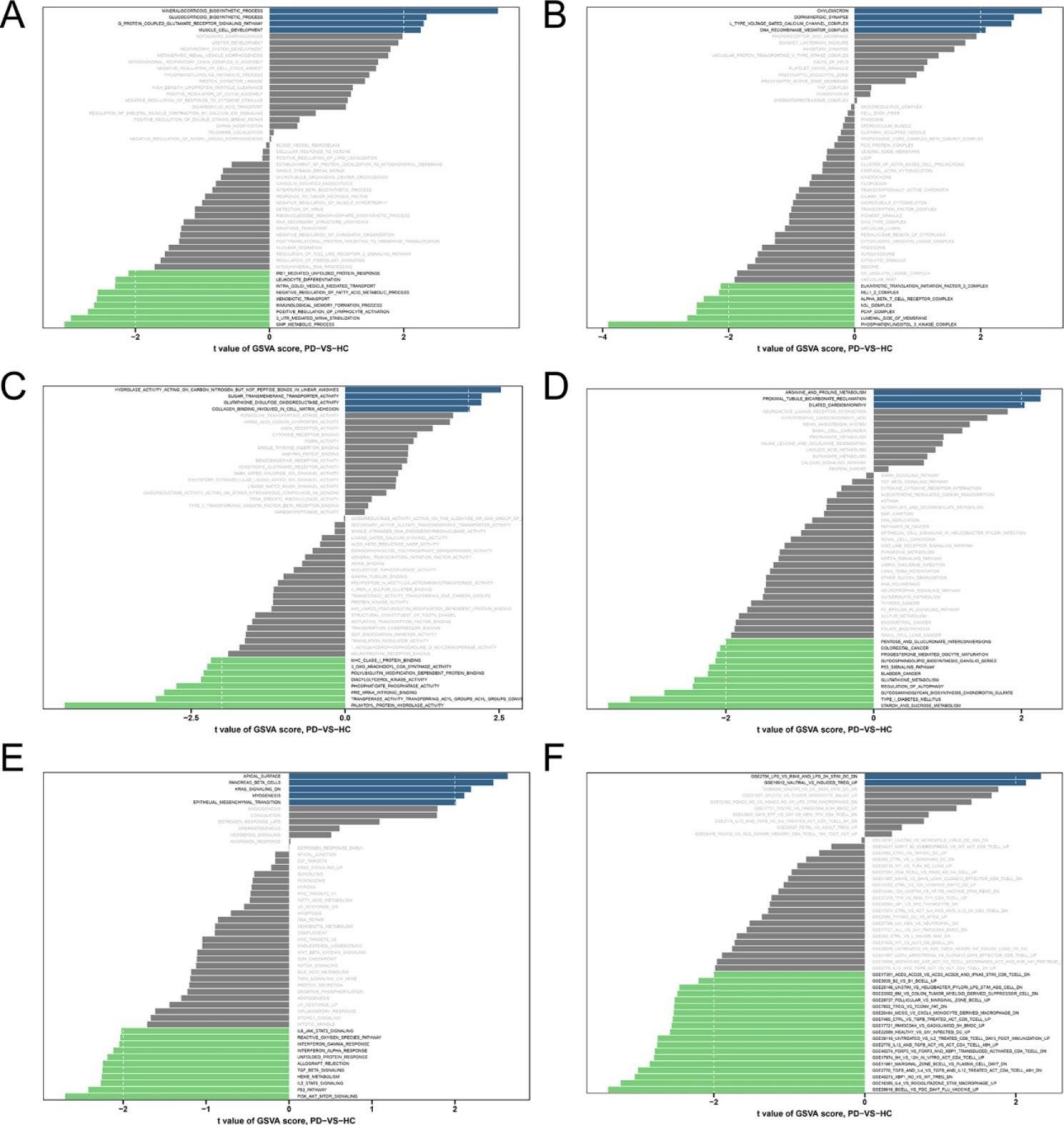
The results of different reference gene sets of GSVA. **A** GO-BP gene sets. **B** GO-CC gene sets. **C** GO-MF gene sets. **D** KEGG gene sets. **E** Hallmarker gene sets. **F** Immunologic signatures gene sets

We performed GSEA and GSVA analysis to screen biological differences between PD and HC. The enrichment analysis results of GO-BP, GO-CC and GO-MF were displayed (Fig. 4A, B, C). When KEGG and hallmark gene sets as the reference sets, the GSVA enrichment analysis revealed that PI3K-AKT-mTOR signaling, reactive oxygen species pathway, P53 signaling pathway and regulation of autophagy were involved in the pathogenesis of PD (Fig. 4D, E). We also found some related immunologic pathways significantly enriched between PD and HC (Fig. 4F). In addition, GSEA analysis of KEGG pathway uncovered some underlying pathways in PD (Table 1), such as autophagy, apoptosis, necroptosis, NOD-like receptor signaling pathway, TNF signaling pathway, ubiquitin mediated proteolysis, cellular senescence, mitophagy, Parkinson disease, alcoholic liver disease and neutrophil extracellular trap formation.

**Table 1:** The KEGG pathway of GSEA analysis

ID	Description	Set size	Enrichment core	NES	Rank
hsa04140	Autophagy - animal	127	-0.488	-1.840	3669
hsa04145	Phagosome	127	-0.423	-1.594	2572
hsa05169	Epstein-Barr virus infection	187	-0.524	-2.050	2794
hsa04062	Chemokine signaling pathway	173	-0.454	-1.766	2868
hsa04210	Apoptosis	130	-0.432	-1.634	3326
hsa04621	NOD-like receptor signaling pathway	162	-0.423	-1.638	2793
hsa04910	Insulin signaling pathway	130	-0.443	-1.677	3804
hsa04514	Cell adhesion molecules	128	-0.464	-1.749	1477
hsa04218	Cellular senescence	144	-0.459	-1.756	3332
hsa04142	Lysosome	125	-0.515	-1.929	3342
hsa05206	MicroRNAs in cancer	159	-0.454	-1.751	3033
hsa04936	Alcoholic liver disease	134	-0.424	-1.608	2460
hsa05152	Tuberculosis	165	-0.494	-1.916	2480
hsa05161	Hepatitis B	155	-0.510	-1.962	3092
hsa05162	Measles	134	-0.520	-1.970	2403
hsa04613	Neutrophil extracellular trap formation	102	-0.556	-2.039	3033
hsa04071	Sphingolipid signaling pathway	111	-0.498	-1.840	2794
hsa04650	Natural killer cell mediated cytotoxicity	110	-0.554	-2.041	3065
hsa04668	TNF signaling pathway	110	-0.534	-1.967	3122
hsa04931	Insulin resistance	104	-0.515	-1.886	3804
hsa04144	Endocytosis	222	-0.361	-1.429	2580
hsa04120	Ubiquitin mediated proteolysis	130	-0.398	-1.505	3719
hsa04620	Toll-like receptor signaling pathway	94	-0.435	-1.577	3316
hsa04080	Neuroactive ligand-receptor interaction	342	0.358	1.677	3930
hsa05012	Parkinson disease	220	0.317	1.416	1331
hsa04217	Necroptosis	122	-0.391	-1.465	3016
hsa01200	Carbon metabolism	105	-0.408	-1.495	1901
hsa04137	Mitophagy - animal	65	-0.444	-1.501	3894
hsa04730	Long-term depression	56	-0.465	-1.533	3459

### Candidate genes selected by machine learning methods

**Fig. 5:**
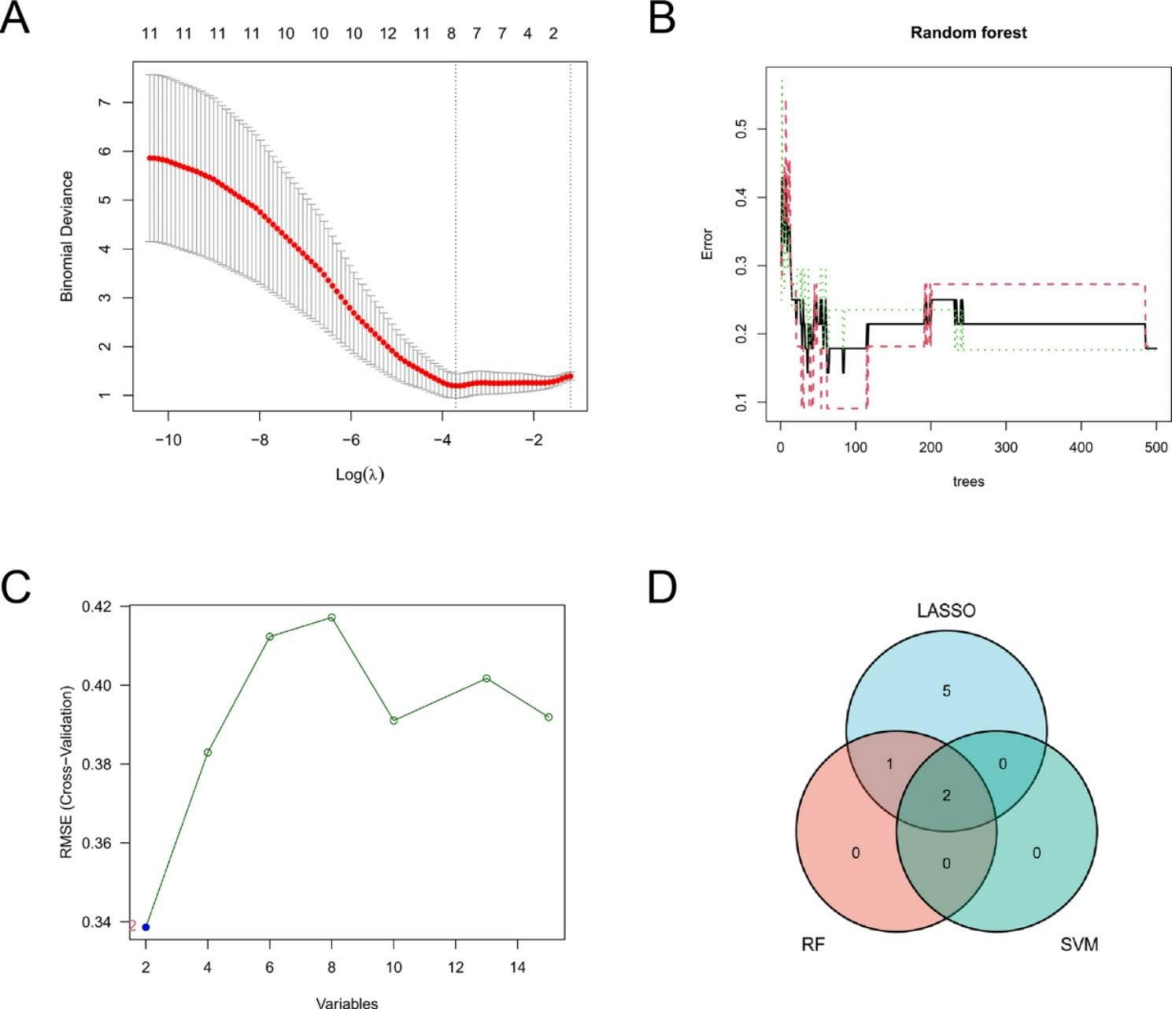
Identification of candidate genes associated with diagnosis using the machine learning method. **A** LASSO regression analysis. **B** Random Forest. **C** Support Vector Machine. **D** Venn diagram for screened candidate genes between LASSO, RF and SVM. RF: random forest; SVM: support vector machine; LASSO: least absolute shrinkage and selection operator analysis

**Fig. 6:**
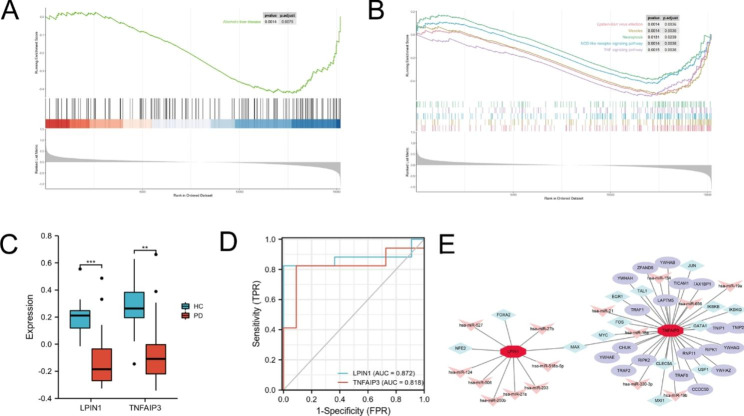
The KEGG pathway and the validation in GSE18838 dataset of candidate genes. **A** The plot showing the KEGG pathways enriched by LPINI. **B** The plot showing the KEGG pathways enriched by TNFAIP3. **C** The expression levels of LPINI and TNFAIP3 in GSE18838. **D** The ROC curve of two candidate genes. **E** The TF-miRNA coregulatory network of LPINI and TNFAIP3. Circle represents protein, diamond represent transcription factor (TF), and arrow represent miRNA. ** p < 0.01, ***p < 0.001

We used LASSO logistic regression algorithm to identify 8 genes from 15 ferroptosis-related-WGCNA genes as key biomarkers for PD (Fig. 5A), RF and SVM-RFE algorithm were also used to screen candidate genes (Fig. 5B, C). Overlapped genes obtained via three algorithms were considered as candidate biomarkers, and finally two genes, LPINI and TNFAIP3 were attained as the biomarkers (Fig. 5D). The KEGG pathway of GSEA analysis on two characteristic genes were shown (Fig. 6A, B). LPINI involved in alcoholic liver disease and TNFAIP3 mainly related to Epstein-Barr virus infection, measles, necroptosis, NOD-like receptor signaling pathway and TNF signaling pathway. In order to further test the diagnostic efficacy of LPINI and TNFAIP3 for PD, we analyzed the expression levels and validated with the GSE18838 microarray expression matrix. Then we found the two genes were downregulated in PD whole blood and ROC curve indicated that they had better diagnostic potential, the AUC is 0.872 (95% CI: 0.723-1.000) and 0.818 (95% CI: 0.647–0.989) for LPINI and TNFAIP3, respectively (Fig. 6C, D). Moreover, GSE72267 was treated as a validation data set including 40 PD patients and 20 healthy controls (Additional file 4: Figure S4). The TF-miRNA coregulatory network of LPIN1 and TNFAIP3 was established on NetworkAnalyst (Fig. 6E).

### GO and KEGG analysis

**Fig. 7:**
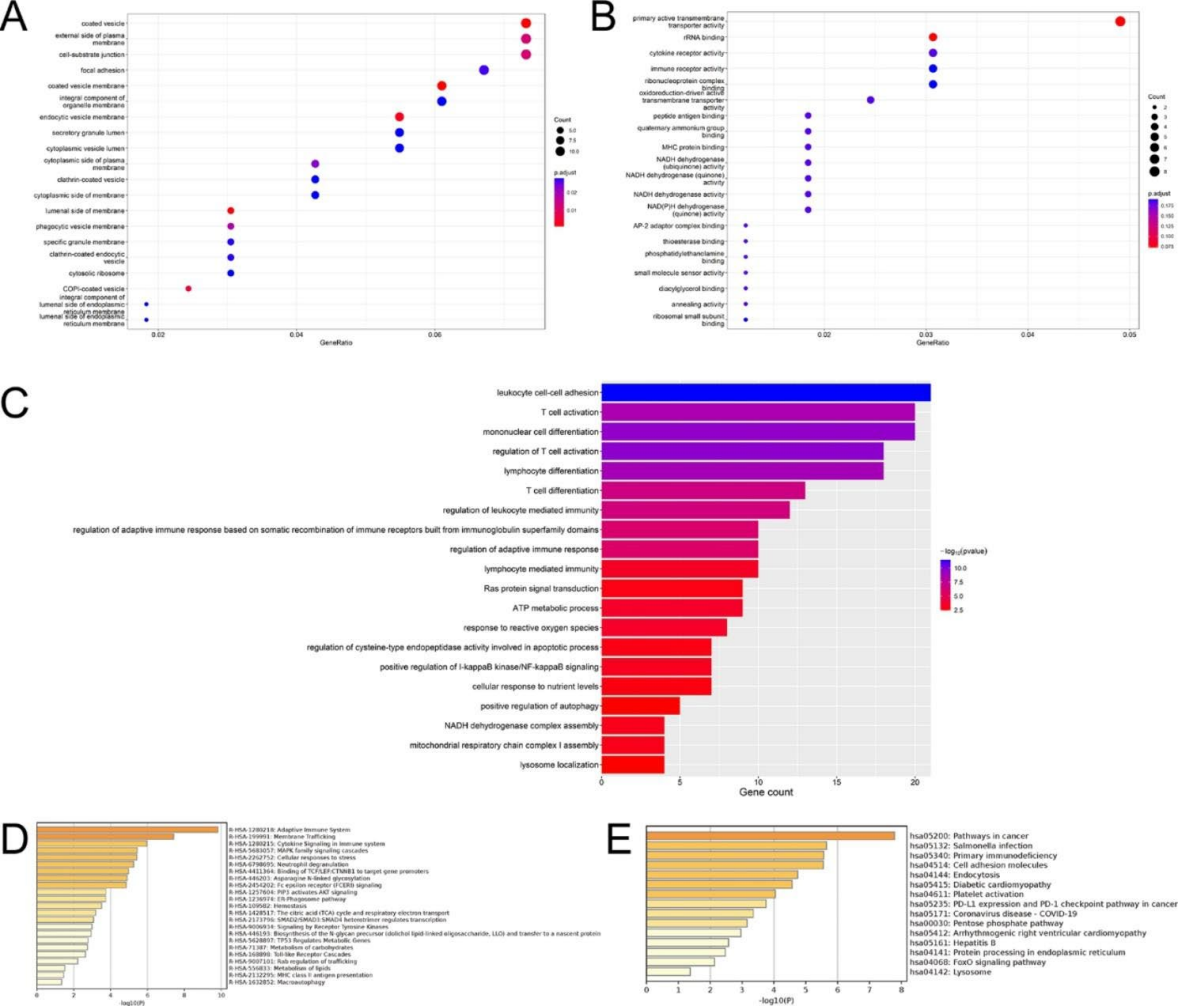
GO and KEGG pathway enrichment results of WGCNA-DEGs. **A, B, C** The analysis of GO_CC, GO_MF and GO_BP. **D, E** The Reactome and KEGG pathway

GO analysis was performed to illustrate the functional annotations of 179 WGCNA-DEGs. The result of cell composition for GO analysis was shown in Fig. 7A. The most enriched GO terms in the biological process category were mitochondrial respiratory complex I assembly, positive regulation of autophagy, response to reactive oxygen species and T cell activation/differentiation, and in the molecular function category were NADH dehydrogenase (ubiquinone) activity, MHC protein binding, immune receptor activity, and ATP metabolism process, Ras protein signal transduction, response to reactive oxygen species and positive regulation of I-kappaB kinase/NF-kappaB signaling and so on (Fig. 7B, C). KEGG and Reactome analysis was conducted to investigate the related signaling pathways. Among the Reactome pathways, macroautophagy, MHC class II antigen presentation, metabolism of lipids, toll-like receptor cascades and cellular responses to stress were involved in PD (Fig. 7D). In addition, KEGG pathway analysis also revealed that lysosome, FoxO signaling pathway, diabetic cardiomyopathy and PD-L1 expression and PD-1 checkpoint pathway in cancer may related to PD (Fig. 7E).

For 15 ferroptosis-WGCNA genes, the significantly enriched GO terms indicated that cellular response to TOR signaling, signaling transduction by p53 class mediator, selective autophagy, response to active oxygen species or metal ion or oxidative stress, fatty acid metabolic process and neuron death were associated with ferroptosis and PD (Table 2). The KEGG results suggest that mTOR signaling pathway, cellular senescence, neutrophil extracellular trap formation, pathways of neurodegeneration-multiple diseases, NF-kappa B signaling pathway and so on, which may play an important role in PD (Table 3).

**Table 2:** The enriched terms of Gene ontology for ferroptosis-WGCNA genes

Term	ID	Description	p.adjust	Gene ID
BP	GO:0007568	aging	7.92E-04	MTOR/ATG7/MAPK8/ATM/MAPK3
BP	GO:0000422	autophagy of mitochondrion	2.35E-03	HIF1A/ATG7/ATG13
BP	GO:0008366	axon ensheathment	3.99E-02	MTOR/LPIN1
BP	GO:0007569	cell aging	6.02E-03	MTOR/MAPK8/ATM
BP	GO:0036473	cell death in response to oxidative stress	2.63E-02	HIF1A/ATG7
BP	GO:0071347	cellular response to Interleukin-1	3.18E-02	HIF1A/MAPK3
BP	GO:0090398	cellular senescence	2.60E-02	MAPK8/ATM
BP	GO:0006631	fatty acid metabolic process	3.06E-02	MTOR/LPIN1/MAPK3
BP	GO:0007612	learning	4.27E-02	MTOR/HIF1A
BP	GO:0002260	lymphocyte homeostasis	1.69E-02	HIF1A/TNFAIP3
BP	GO:0016236	macroautophagy	2.90E-05	MTOR/HIF1A/ATG7/ATG13/MAPK8/MAPK3
BP	GO:0070997	neuron death	2.74E-02	MTOR/HIF1A/ATG7
BP	GO:0034250	positive regulation of cellular amide metabolic process	4.97E-02	MTOR/MAPK3
BP	GO:0010506	regulation of autophagy	3.60E-05	MTOR/HIF1A/ATG7/MAPK8/ATM/MAPK3
BP	GO:0001959	regulation of cytokine-mediated signaling pathway	4.58E-02	HIF1A/TNFAIP3
BP	GO:0035303	regulation of dephosphorylation	3.81E-02	MTOR/LPIN1
BP	GO:0051090	regulation of DNA-binding transcription factor activity	3.85E-02	MAPK8/TNFAIP3/MAPK3
BP	GO:0010821	regulation of mitochondrion organization	4.27E-02	HIF1A/MAPK8
BP	GO:0031644	regulation of nervous system process	4.27E-02	MTOR/LPIN1
BP	GO:1,903,203	regulation of oxidative stress-induced neuron death	7.98E-03	HIF1A/ATG7
BP	GO:0031396	regulation of protein ubiquitination	1.18E-02	MTOR/TNFAIP3/HERPUD1
BP	GO:0010038	response to metal ion	2.86E-02	HIF1A/MAPK8/MAPK3
BP	GO:0006979	response to oxidative stress	2.15E-04	HIF1A/ATG7/MAPK8/ABCC1/TNFAIP3/MAPK3
BP	GO:0000302	response to reactive oxygen species	2.11E-03	HIF1A/MAPK8/TNFAIP3/MAPK3
BP	GO:0061912	selective autophagy	1.95E-02	ATG13/MAPK3
BP	GO:0072331	signal transduction by p53 class mediator	9.24E-03	MTOR/CD44/ATM
BP	GO:0031929	TOR signaling	5.64E-03	MTOR/HIF1A/ATM
CC	GO:0000407	phagophore assembly site	2.29E-02	ATG7/ATG13
MF	GO:0004707	MAP kinase activity	5.86E-03	MAPK8/MAPK3
MF	GO:0106310	protein serine kinase activity	7.56E-03	MTOR/MAPK8/ATM/MAPK3
MF	GO:0004674	protein serine/threonine kinase activity	7.56E-03	MTOR/MAPK8/ATM/MAPK3

**Table 3:** List of top enriched KEGG pathways of ferroptosis-WGCNA genes

ID	Description	GeneRatio	BgRatio	p.adjust	Gene ID
hsa04140	Autophagy - animal	6/14	141/8149	1.05E-05	2475/3091/10,533/9776/5599/5595
hsa04930	Type II diabetes mellitus	3/14	46/8149	1.58E-03	2475/5599/5595
hsa04012	ErbB signaling pathway	3/14	85/8149	4.40E-03	2475/5599/5595
hsa05235	PD-L1 expression and PD-1 checkpoint pathway in cancer	3/14	89/8149	4.54E-03	2475/3091/5595
hsa04657	IL-17 signaling pathway	3/14	94/8149	5.00E-03	5599/7128/5595
hsa04066	HIF-1 signaling pathway	3/14	109/8149	6.85E-03	2475/3091/5595
hsa04668	TNF signaling pathway	3/14	112/8149	7.02E-03	5599/7128/5595
hsa04071	Sphingolipid signaling pathway	3/14	119/8149	7.58E-03	5599/4363/5595
hsa04068	FoxO signaling pathway	3/14	131/8149	9.14E-03	5599/472/5595
hsa04210	Apoptosis	3/14	136/8149	9.57E-03	5599/472/5595
hsa04910	Insulin signaling pathway	3/14	137/8149	9.57E-03	2475/5599/5595
hsa04150	mTOR signaling pathway	3/14	156/8149	1.24E-02	2475/23,175/5595
hsa04218	Cellular senescence	3/14	156/8149	1.24E-02	2475/472/5595
hsa05010	Alzheimer disease	4/14	384/8149	1.85E-02	2475/9776/5599/5595
hsa04621	NOD-like receptor signaling pathway	3/14	184/8149	1.85E-02	5599/7128/5595
hsa04613	Neutrophil extracellular trap formation	3/14	190/8149	1.96E-02	2475/10,533/5595
hsa05169	Epstein-Barr virus infection	3/14	202/8149	2.26E-02	960/5599/7128
hsa05208	Chemical carcinogenesis - reactive oxygen species	3/14	223/8149	2.57E-02	3091/5599/5595
hsa04920	Adipocytokine signaling pathway	2/14	69/8149	2.57E-02	2475/5599
hsa04137	Mitophagy - animal	2/14	72/8149	2.59E-02	3091/5599
hsa05022	Pathways of neurodegeneration - multiple diseases	4/14	476/8149	2.62E-02	2475/9776/5599/5595
hsa04658	Th1 and Th2 cell differentiation	2/14	92/8149	3.55E-02	5599/5595
hsa04933	AGE-RAGE signaling pathway in diabetic complications	2/14	100/8149	3.78E-02	5599/5595
hsa04064	NF-kappa B signaling pathway	2/14	104/8149	3.78E-02	7128/472
hsa04620	Toll-like receptor signaling pathway	2/14	104/8149	3.78E-02	5599/5595
hsa04660	T cell receptor signaling pathway	2/14	104/8149	3.78E-02	5599/5595
hsa05016	Huntington disease	3/14	306/8149	3.92E-02	2475/9776/5599
hsa04931	Insulin resistance	2/14	108/8149	3.92E-02	2475/5599
hsa04722	Neurotrophin signaling pathway	2/14	119/8149	4.53E-02	5599/5595

### Estimation of infiltrating immune cells and correlation analysis

**Fig. 8:**
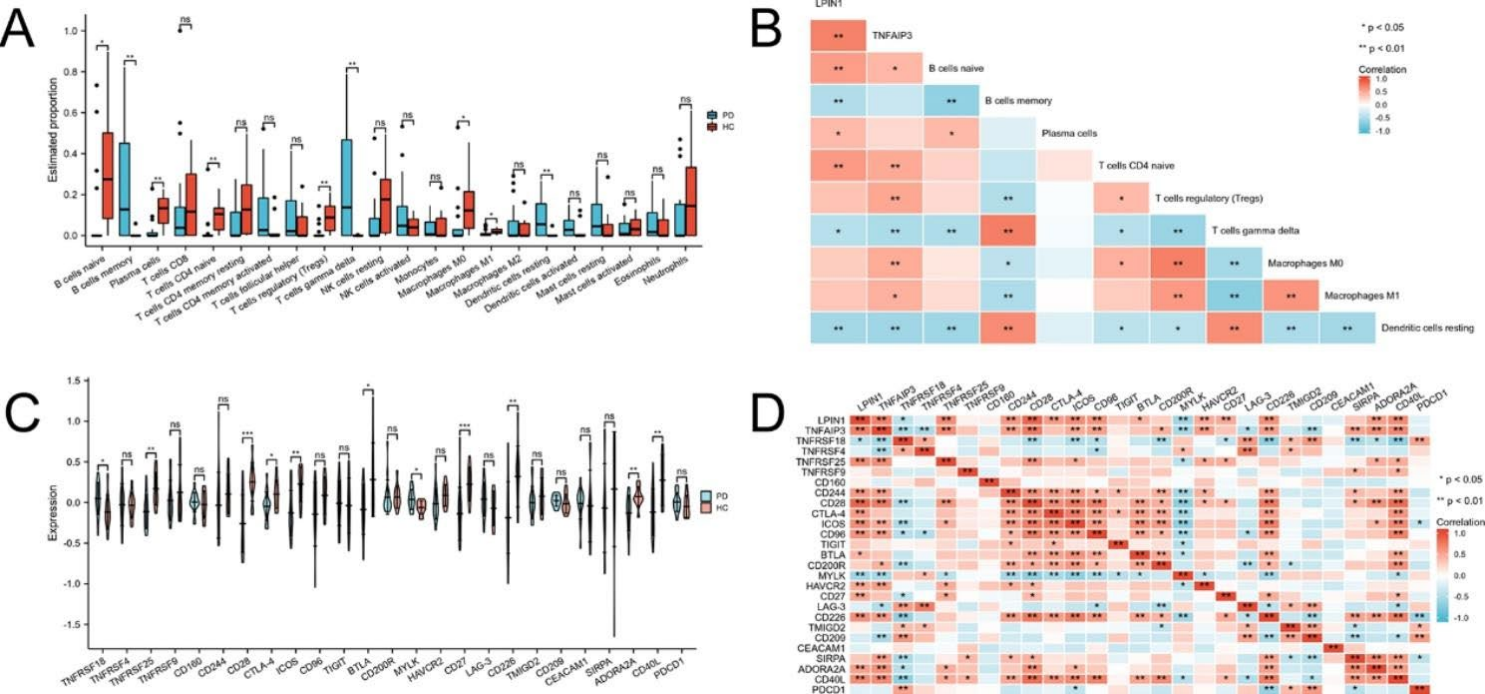
The status of immune cell infiltration and expression of immune checkpoint genes. **A** Boxplots comparing the proportions of 22 major immune cell subsets between PD and HC samples. **B** Correlation between LPINI, TNFAIP3, and infiltrating immune cells by CIBERSORT. **C** The expression of immune checkpoint genes between PD and HC samples. **D** Correlation between LPINI, TNFAIP3, and ICGs.

Firstly, we estimated the proportion of 22 infiltrating immune cells using the gene matrix of 28 samples by “CIBERSORT” algorithm. Compared to the results for HC, the proportions of naïve B cells, plasma cells, naïve CD4 T cells, regulatory T cells, macrophages M0, and macrophages M1 were significantly lower in the PD samples, while the proportions of memory B cells, gamma delta T cells, and resting dendritic cells were significantly higher (Fig. 8A). Positive and negative relationships between candidate genes and infiltrating immune cells were all discovered via spearman analysis. LPINI had positive correlation with naïve B cells, plasma cells and naïve CD4 T cells, while had negative correlation with memory B cells, gamma delta T cells and resting dendritic cells. TNFAIP3 had positive correlation with naïve B cells, naïve CD4 T cells, regulatory T cells, macrophages M0 and macrophages M1, while had negative correlation with gamma delta T cells and resting dendritic cells (Fig. 8B).

In addition, for immune checkpoint genes expressed on T cells, TNFRSF18, TNFRSF25, CD28, CTLA-4, ICOS, BTLA, MYLK, CD27, CD226, ADORA2A and CD40L were different significantly between two groups (Fig. 8C). For correlation analysis between candidate genes and immune checkpoint genes, which were displayed on Fig. 8D. LPIN1 had significant correlations with all the above different ICGs, however, TNFAIP3 only was correlated with TNFRSF18, TNFRSF25, CD28, ICOS, MYLK, CD226, ADORA2A and CD40L.

### Demographic and clinical characteristics of the PD patients and healthy controls

Demographic characteristics of participants are summarized in Table 4, the clinical characteristics of early and middle-advanced PD patients are shown in Table 5. Between the healthy controls and PD patients, RBC, Hb, Hct, the ratio of monocyte and lymphocyte were significantly different (p = 0.000, p = 0.000, p = 0.000, p = 0.031). Furthermore, the difference in WBC, RBC, Hb, Hct were also statistically significant between the HC and early-stage PD patients (p = 0.031, p = 0.000, p = 0.000, p = 0.000) (Table 4). For the comparison of early and middle-advanced stage PD patients, age, disease duration (years), the score of MDS-UPDRS III “off”, WBC, neutrophils (%), lymphocyte (%), the ratio of neutrophils and lymphocyte, the ratio of monocyte and lymphocyte also had statistical difference (p = 0.002, p = 0.000, p = 0.000, p = 0.026, p = 0.003, p = 0.001, p = 0.002, p = 0.031) (Table 5). There was a significant difference in the UPDRS score between the early and middle-advanced stage of PD, which was in line with the disease degree of two stages.

**Table 4:** Demographic and clinical characteristics of the PD patients and healthy controls used in this study

Variable	PD (n = 70)	HC (n = 39)	*p* value
Age, years	65.42(8.85)	63.38(9.03)	0.254
Male/female ratio	41/29	25/14	0.571
Disease duration, years	7.16(3.94)		
H&Y stage, off (1|1.5|2|2.5|3|4|5|)	5|7|10|8|28|10|2		
MDS-UPDRS III “off” (0-132)	48.94(18.64)		
WBC, 10^9/L	6.09(2.09)	6.29(1.15)	0.064
Neutrophils (%)	62.28(10.90)	59.03(6.63)	0.202
Lymphocyte (%)	29.14(9.66)	32.18(6.77)	0.084
Monocytes (%)	6.19(2.15)	6.02(1.35)	0.972
N/L	2.87(2.94)	1.97(0.68)	0.121
M/L	0.24(0.15)	0.19(0.07)	0.031
RBC, 10^12^/L	4.37(0.48)	4.78(0.40)	0.000
Hb, g/L	133.97(13.98)	145.38(11.76)	0.000
Hct, L/L	0.402(0.04)	0.440(0.033)	0.000

Values are means ± SD unless otherwise stated. PD, Parkinson’s disease; HC, healthy controls; H&Y stage, Hoehn and Yahr scale; MDS-UPDRS III, Movement Disorders Society-Unified Parkinson Disease Rating Scale, motor part; N/L, neutrophils/lymphocyte; M/L, monocytes/ lymphocyte

**Table 5:** Demographic and clinical characteristics of early and middle-advanced PD patients

Variable	Early (n = 30)	Mid-advanced (n = 40)	*p* value
Age, years	61.60(9.58)	68.30(7.12)	0.002
Male/female ratio	16/14	25/15	0.441
Disease duration, years	5.28(3.46)	8.58(3.72)	0.000
MDS-UPDRS III “off” (0-132)	38.97(15.70)	56.43(17.24)	0.000
WBC, 10^9/L	5.51(1.79)	6.52(2.21)	0.026
Neutrophils (%)	57.89(10.40)	65.57(10.20)	0.003
Lymphocyte (%)	33.34(9.51)	25.99(8.60)	0.001
Monocytes (%)	6.71(2.68)	5.81(1.57)	0.146
N/L	2.41(3.47)	3.21(2.46)	0.002
M/L	0.24(0.20)	0.24(0.08)	0.031
RBC, 10^12/L	4.38(0.48)	4.37(0.49)	0.927
Hb, g/L	133.27(14.68)	134.5(13.59)	0.718
Hct, L/L	0.400(0.041)	0.403(0.039)	0.739

Values are means ± SD unless otherwise stated. PD, Parkinson’s disease; HC, healthy controls; H&Y stage, Hoehn and Yahr scale; MDS-UPDRS III, Movement Disorders Society-Unified Parkinson Disease Rating Scale, motor part; N/L, neutrophils/lymphocyte; M/L, monocytes/ lymphocyte

### Plasmic levels of LPIN1 and TNFAIP3 in PD patients and healthy controls

**Fig. 9:**
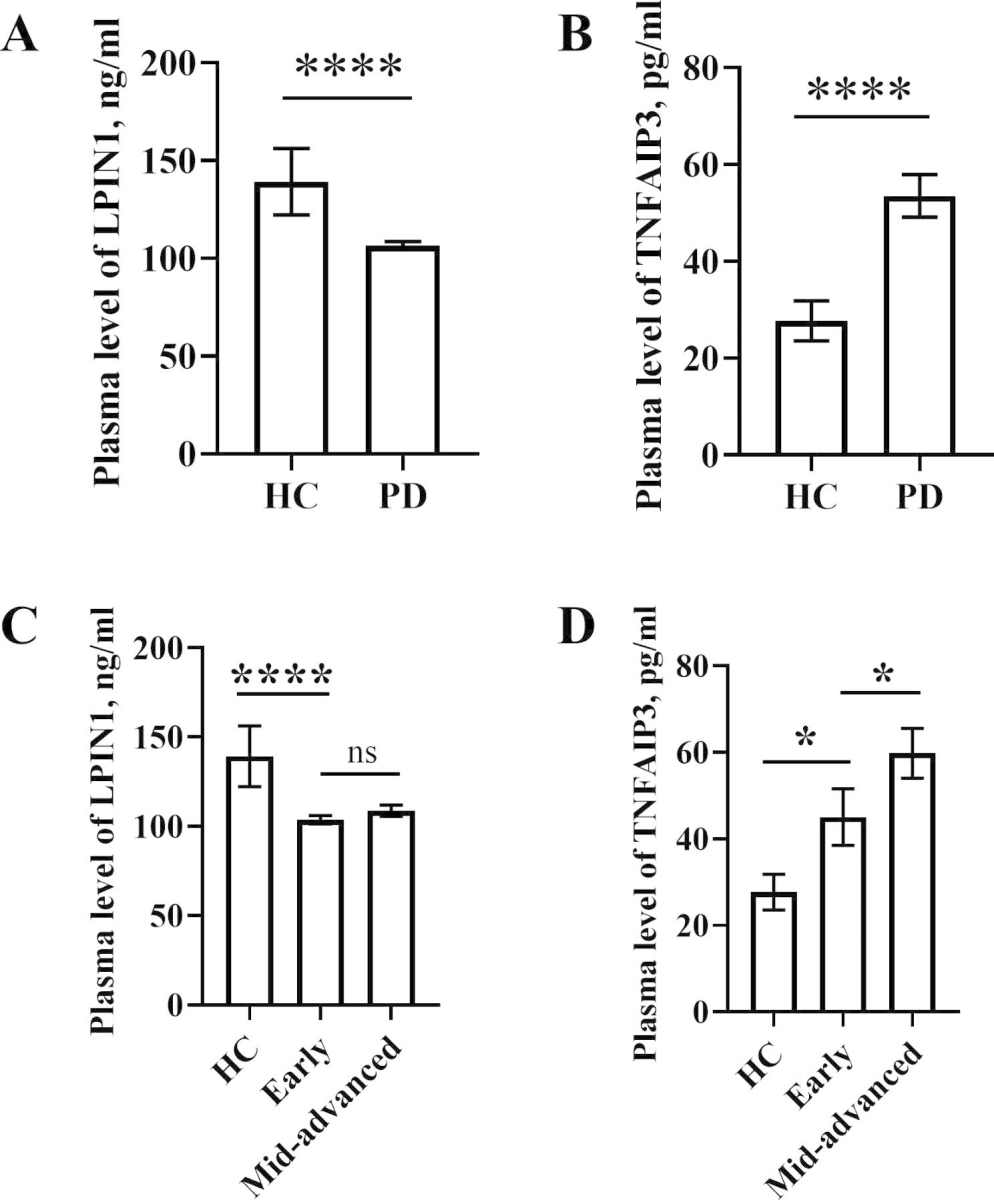
The ELISA verification of two biomarkers. **A, B** The plasma level of LPIN1 and TNFAIP3 in HC and PD. **C, D** The plasma level of LPIN1 and TNFAIP3 in HC, early and middle-advanced PD patients. HC: healthy controls; PD: Parkinson’s disease; early: early stage; mid-advanced: middle and advanced stage. *p < 0.05, ****p < 0.0001. ns, no significance

The plasmic concentration of LPIN1 in patients with PD (105.7 ng/mL [range 56.98 to 161.3 ng/mL]) was significantly lower than that in HC (121.0 ng/mL [range 87.03 to 773.4 ng/mL]) (p < 0.0001) (Fig. 9A). While there was a significant increase of TNFAIP3 plasma concentration in PD patients (45.91 pg/ml [range 4.61 to 193.9 pg/ml]) compared with HC (20.50 pg/ml [range 5.84 to 159.5 pg/ml]) (p < 0.0001) (Fig. 9B). When the PD patients were divided into early stage and middle-advanced stage, the plasma level of LPIN1in early stage PD (101.7 ng/mL [range 77.96 to 137.7 ng/mL]) was significantly lower than that in HC (p < 0.0001), while there was no statistically significant difference between early and middle-advanced stage PD patients (110.0 ng/mL [range 56.98 to 161.3 ng/mL]) (p = 0.2806) (Fig. 9C). A significant elevation of TNFAIP3 level in early stage PD patients (35.06 pg/mL [range 4.61 to 135.2 pg/mL]) compared with HC was found (p = 0.0407), as well as there was also significant difference between early stage and middle-advanced stage PD patients (50.63 pg/mL [range 7.75 to 193.9 pg/mL]) (p = 0.0459) (Fig. 9D). Furthermore, a correlation plot between the expression levels of two molecules and clinical parameter was shown in Additional file 5: Figure S5, TNFAIP3 had a weak correlation with age, basophil, Hoehn and Yahr scale, disease stage.

### Diagnostic value of plasmic LPIN1 and TNFAIP3 in PD

**Fig. 10:**
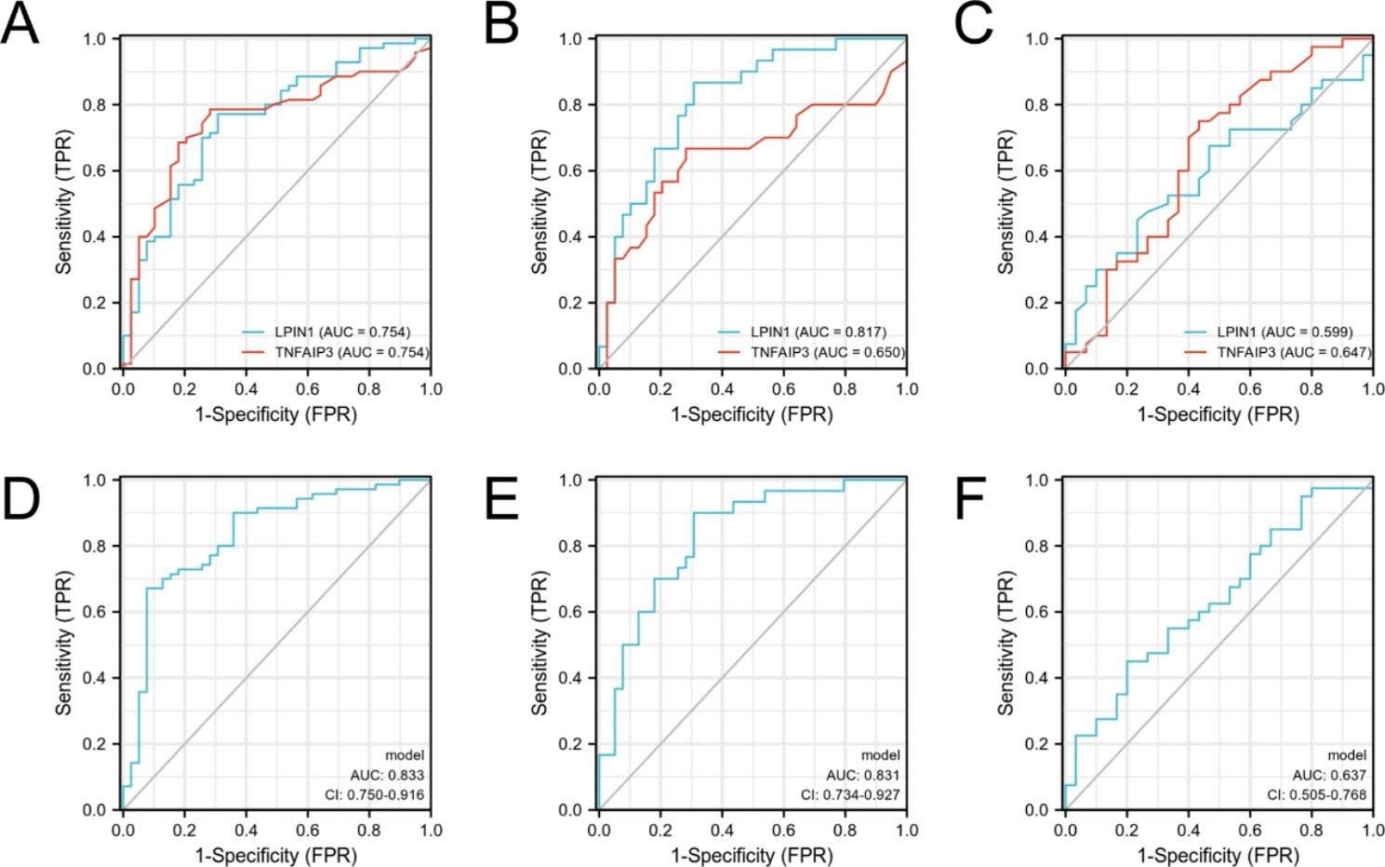
The ROC of two biomarkers. **A, B, C** Each biomarker plot one ROC (HC vs. PD, HC vs. early PD, early vs. middle-advanced PD). **D, E, F** Two biomarkers combined using binary logistic regression model (HC vs. PD, HC vs. early PD, early vs. middle-advanced PD). sensitivity (true positive rate) and 1-specificity (false positive rate); AUC: area under curve; CI: 95% confidence interval

Receiver operating characteristic (ROC) curves were applied to evaluate the potential diagnostic value of LPIN1 and TNFAIP3 in PD. The area under ROC curve (AUC) of LPIN1 and TNFAIP3 for PD were 0.754 (95% CI: 0.659–0.849, p < 0.0001, sensitivity = 0.771, specificity = 0.692) and 0.754 (95% CI: 0.660–0.849, p < 0.0001, sensitivity = 0.686, specificity = 0.821) (Fig. 10A) (Additional file 6: Table S6). In distinguishing the early stage PD from HC, the AUC of LPIN1 and TNFAIP3 were 0.817 (95% CI: 0.717–0.917, p < 0.0001, sensitivity = 0.867, specificity = 0.692) and 0.650 (95% CI: 0.507–0.794, p = 0.040, sensitivity = 0.667, specificity = 0.718) (Fig. 10B) (Additional file 7: Table S7). However, LPIN1 and TNFAIP3 don’t performed well in distinguishing the early stage from middle-advanced stage PD patients (LPIN1: AUC = 0.599, 95% CI: 0.465–0.733, p = 0.146; TNFAIP3: AUC = 0.647, 95% CI: 0.510–0.783, p = 0.035) (Fig. 10C) (Additional file 8: Table S8). Then, we used logistic regression analysis and the results indicated that LPIN1 and TNFAIP3 performed better in combination for prediction (HC vs. PD, AUC = 0.833, 95% CI: 0.750–0.916, p < 0.0001; HC vs. early PD, AUC = 0.831, 95% CI: 0.734–0.927, p < 0.0001) (Fig. 10D, E), while the diagnostic efficacy was relatively poor in discriminating early and middle-advanced PD (AUC = 0.637, 95% CI: 0.505–0.768, p = 0.041) (Fig. 10F).

## Discussion

Herein, we performed WGCNA analysis, intersected between DEGs, FRGs and interesting module, then identified 15 ferroptosis-related WGCNA genes and 179 WGCNA-DEGs genes. Enrichment analysis including GSVA, GSEA, GO and KEGG were operated. LPINI and TNFAIP3, as candidate genes, were determined by machine learning method (LASSO, SVM and RF). Moreover, LPINI and TNFAIP3 were differently expressed in the plasma of PD patients and healthy controls detected by ELISA. With the estimation of infiltrating immune cells and correlation analysis, we found the FRGs was associated with ICGs, immune regulation. In addition, ROC curve indicated that LPINI and TNFAIP3 may provide a novel diagnostic biomarker for PD. These results demonstrated that candidate genes might participate in the processes of regulating immune cell infiltration and immune checkpoint genes expression in PD.

Aging is a major risk factor for various neurodegenerative disorders and accompany with gently accumulation of iron in the brain that relates with lipid peroxidation and reactive oxygen species production that represents the state of oxidative stress [5]. Iron can upregulate the levels of α-synuclein, amyloid precursor protein (APP) and amyloid β-peptide (Aβ) [5]. Selective deposition of iron in SN is one of the essential pathogenic factors [12], glutathione (GSH) loss in SN and oxidative stress are predispositions to PD [5]. In addition, recent emerging evidence suggests that ferroptosis is a prevalent cell death pathway for dopaminergic neurons [16]. For example, iron accumulation in aging glial cells could impair neurons by increasing proinflammatory factors to establish neuroinflammation [26]. Ferroptosis is defined as Fe (II)-dependent regulated necrosis accompanied lipid peroxidation [27], a mitochondria-dependent type of cell death [28], which was an important cell death pathway in Lund human mesencephalic cells, these had been confirmed ex vivo (in organotypic slice cultures) and in vivo (in the MPTP mouse model of PD) [16]. A study found that when SH-SY5Y human neuroblastoma cells were treated with PQ (paraquat dichloride) and Fer-1 (a specific inhibitor of ferroptosis) together, Fer-1 could inhibit the production of lipid reactive oxygen species and ameliorate ferroptosis by upregulating the expression of GPX4 (glutathione peroxidase 4) and SLC7A11 (cystine/glutamate antiporter). Fer-1 also inhibited the accumulation of ferrous iron in mitochondria, protected against PQ-induced damage, and maintained mitochondrial integrity [29]. Moreover, mounting studies have shown that potential physiological roles of ferroptosis in cancer, ischemia/reperfusion injuries, neurodegeneration and other pathological conditions, nevertheless the exact contribution of ferroptosis to these pathologies is unclear [5].

Lipin1 is a Mg^2+^-dependent phosphatidic acid phosphatase (PAP) enzyme closely related to glycolipid metabolism, produced by the expression of LPIN1 [30], referred as a member of the lipin family, which converts phosphatidic acid (PA) to diacylglycerol (DAG), a precursor of triacylglycerol and phospholipids [30, 31]. Additionally, LPIN1 functions as a transcriptional coregulator via directly interacting with nuclear peroxisome proliferator-activated receptor α (PPARα) and PPARα co-stimulatory factor 1 α (PGC1α) to regulate the genes involved in fatty acid oxidation [32]. It is reported that LPIN1 can promote several processes, including cell differentiation, inflammation and autophagy [31]. The human lipin1 has three isoforms (lipin1α, lipin1β, lipin1γ) derived from alternative mRNA splicing. Lipin1α and lipin1β are lowly expressed in the brain, conversely, lipin1γ is highly expressed in normal human brain, indicating that lipin1γ may be a specialized regulatory protein in brain lipid metabolism [32, 33]. Latest study confirmed the presence of cognitive impairment in the mice with hippocampus of Lipin1-deficient, including the worsen spatial learning and memory ability, decreased synapse number, reduced protein levels of BDNF, SYP and PSD95. Shang et al. reported that lipin1 impaired synaptic plasticity, disturbed lipid homeostasis, and damaged spatial learning and memory by inhibiting DAG-PKD-ERK signaling pathway in Fld mice (a mutation in the Lpin1 gene) [34]. In another research, authors considered that neuroprotection of LPIN1 was associated with inhibition of the PKD/Limk1/Cofilin signaling pathway, and LPIN1 might ameliorate the cognitive impairments in Diabetic encephalopathy (DE) animal models [35]. The loss of Lipin1 decreases DAG expression, which may lead to lipid metabolism disorders, induce autophagy overaction and promote Diabetic Peripheral Neuropathy (DPN). In contrast, overexpression of Lipin1 can reduce autophagy disorders and alleviate DPN [36]. Autophagy plays an important role in neurodegenerative diseases and nerve tissue injury [37].

A20, also known as TNF-α-induced protein 3 (TNFAIP3), is a ubiquitin editing enzyme with both E3 ubiquitin ligase activity and deubiquitinating enzyme (DUB) activity [38], also functions as a key negative regulator of NF-κB transcription factors and an anti-inflammatory molecule that plays an important part in both immune responses and cell death [39], which can suppress NF-κB signaling downstream from T cell receptor (TCR), B cell receptor (BCR), tumor necrosis factor receptor (TNFR), interleukin 1 receptor (IL-1R), Toll-like receptors (TLRs), NOD-like receptors (NLRs) and so on [40]. NF-κB signaling pathway can activate the innate and adaptive immune system, yet its improper activation indicates the development of chronic inflammation and cell death [41]. Moreover, NF-κB has been implicated in the pathogenesis of a variety of neurodegenerative diseases [42]. TNFAIP3, as a central negative regulator of NF-κB transcription factors by multiple mechanisms, which probably has functions in the regulation of NF-κB signaling in astrocytes and in neurons within the CNS [42]. Microglia A20 deficiency exacerbated multiple sclerosis (MS)-like disease, due to hyperactivation of the NLRP3 inflammasome leading to increased interleukin-1β secretion in mice, suggesting that A20 critically controls microglia activation and inhibits inflammasome-dependent neuroinflammation [43]. After deleting A20 in microglia, CD8 + T cells spontaneously infiltrate the CNS and acquire a viral response signature, also upregulate genes associated with the antiviral response and neurodegenerative diseases [44].

As a regulator of cell death, on the hand, A20 can inhibit TNFα-induced apoptosis through the inhibition of phospholipase A2 and caspase 8 activation, reduce production of reactive oxygen species, diminish collapse of mitochondrial membrane potential, suppress the c-Jun N-terminal kinase and pro-inflammatory cytokines [42]. A20 can also restrict necroptosis in T cells and macrophages via its deubiquitinating motif [38]. On the other hand, A20 may have a proapoptotic function and restrict cell survival, probably due to upregulation of NF-κB-dependent antiapoptotic proteins Bcl-2 and Bcl-x [38]. A20 has been shown to promote survival of CD4 + T cells by restricting the ubiquitylation-dependent activation of mTOR and promoting autophagy [45]. Gradually, depending on the cell type and activated signaling pathway, more evidence indicates that A20 can indirectly counteract inflammatory response by protecting cells from death, which largely dependents on its ubiquitin-binding properties [38, 46].

In the present study, when KEGG and hallmark gene sets as the reference sets, the GSVA enrichment analysis revealed that reactive oxygen species pathway, p53 pathway and regulation of autophagy were involved in the pathogenesis of PD (Fig. 4D, E). For the KEGG analysis of GSEA, we found some pathways including autophagy–animal, apoptosis, NOD-like receptor signaling pathway, cellular senescence, lysosome, Parkinson disease, necroptosis and so on (Table 1). Furthermore, LPIN1 and TNFAIP3 were also involved in the regulation of mentioned signal pathway.

Lastly, we performed immune infiltration analysis on the peripheral blood microarray expression matrix of PD and compared the expression of immune checkpoint genes related to T cells, then revealed that the proportion of immune cells and expression of ICGs were significantly different between two groups. Previous work has started to elucidate the complex effects of ferroptosis on different aspects of the immune function [47]. on the one hand, ferroptosis affects the number and function of immune cells. On the other hand, ferroptotic cells can be recognized by immune cells and then trigger a series of specific inflammatory responses. Furthermore, ferroptosis of immune cells may destroy immune response, and ferroptosis of non-immune cells may cause the release of DAMPs (danger-associated molecular patterns) that induces immune activation [47]. As a programmed necroptosis, ferroptosis is inherently more immunogenic than apoptosis and results in the release of inflammatory cytokines, leading to necro-inflammatory response, which can drive the pro-inflammatory state in certain biological contexts [48]. Because of its high metabolic activity, brain tissue is particularly susceptible to oxidative stress that is a hallmark of various neurodegenerative disorders [49]. Cells under oxidative stress may release immunogenic molecules that triggers a systemic immune response, ultimately leading to cell necrosis [48]. In line with the above mentioned, the specific necrotic signaling pathway of ferroptosis may produce pathogenic cytokines peroxides that impairs the immune response via activating immune cells [48].

In addition, our experiment showed that LPIN1 was under-expressed and TNFAIP3 was upregulated in the plasma of PD patients that was consistent to the validation in GSE72267 (Additional file 4: Figure S4). A previous real-time PCR assay also showed decreased TNFAIP3 expression in PD whole blood samples [50], while in the GSE18838 microarray expression matrix, LPIN1 and TNFAIP3 both were downregulated in PD whole blood. Each biomarker alone could discriminate the PD and HC (AUC > 0.75), however, TNFAIP3 didn’t performed well in distinguishing the early PD from healthy controls (LPIN1: AUC = 0.817, false positive rate = 0.308, false negative rate = 0.133; TNFAIP3: AUC = 0.650, false positive rate = 0.282, false negative rate = 0.333). The diagnostic model formed by the combination of two biomarkers had an AUC of 0.833 (sensitivity = 0.671, specificity = 0.923) in distinguishing PD from HC and an AUC of 0.831 (sensitivity = 0.900, specificity = 0.692) in distinguishing the early PD from HC.

In this study, there are still some limitations. Firstly, The TNFAIP3 levels are inconsistent in different population samples, numerous variables can lead to the inconsistent results, such as choices of assays, methods of sample acquisition, drug treatment, disease severity. Besides, existing clinical information remains incomplete, and validation is required at the genetic level of clinical samples by multiple methods. Therefore, to objectively evaluate the diagnostic effects of LPIN1 and TNFAIP3, it is necessary to strictly control the inclusion and exclusion criteria of PD subjects and collect more complete, accurate clinical data to regulate the influence of other miscellaneous variables on experiment.

## Conclusion

In summary, our results confirmed abnormally under-expression or upregulation of LPINI and TNFAIP3 in the PD plasma, ferroptotic cells and circulating immune system responses are implicated in the pathogenesis of PD. Furthermore, ferroptosis-related genes have correlations with immune checkpoint genes, immune infiltration. Thus, this study further improved the understanding of the effect mechanism of ferroptosis on peripheral blood mononuclear cells (mainly including lymphocyte and monocyte). However, the specific mechanism of LPINI and TNFAIP3 regulate ferroptosis and immunity in PD is not clear. More research is needed to explore the biological effects of LPINI and TNFAIP3 on peripheral immune cells and provide reliably clinical diagnostic markers for PD.

## Electronic supplementary material

Below is the link to the electronic supplementary material.

Supplementary Material 1

Supplementary Material 2

Supplementary Material 3

Supplementary Material 4

Supplementary Material 5

Supplementary Material 6

Supplementary Material 7

Supplementary Material 8

## Data Availability

The datasets generated and/or analyzed (GSE18838 and GSE72267) during this study are publicly available in the GEO database (https://www.ncbi.nlm.nih.gov/geo/), the original contributions presented in this study are included in the article/Supplementary material, further inquiries can be directed to the corresponding author.

## Abbreviations

PD: Parkinson’s disease
SNpc: Substantia nigra pars compacta
GSH: Glutathione
ROS: Reactive oxygen species
FAC: Ferric ammonium citrate
WGCNA: Weighted gene co-expression network analysis
GEO: Gene Expression Omnibus
GSVA: Gene set variation analysis
GSEA: Gene Set Enrichment Analysis
MSigDB: The Molecular Signatures Database
GO: Gene Ontology
KEGG: Kyoto Encyclopedia of Genes and Genomes
ELISA: Enzyme-linked immunosorbent assay
ROC: Receiver operating characteristic
