# Supplementary material for: Identification of ferroptosis related biomarkers and immune infiltration in Parkinson’s disease by integrated bioinformatic analysis
Source: BMC Med Genomics. 2023 Mar 14;16:55. doi: 10.1186/s12920-023-01481-3 (PMC10012699; doi:10.1186/s12920-023-01481-3)
Supplement: Supplementary file 1 — Supplementary Material 1 [file 12920_2023_1481_MOESM1_ESM.docx]

Supplemental Table 1 Clinical characteristics of PD and control cohort included in GSE18838.

| Variable | PD (n=32) | HC (n=36) |
| --- | --- | --- |
| Age, years | 64.24±11.350 | 62.64±9.069 |
| Male/female ratio | 15/2 | 3/8 |
| Disease duration, years | 9.35±6.072 |  |
| Dementia（1\|2\|3） | 1\|14\|2 |  |
| Depression（1\|2\|3） | 7\|10\|0 |  |
| Hallucinations（1\|2\|3） | 3\|12\|2 |  |
| Constipation（1\|2\|3） | 4\|4\|9 |  |
| Freezing（1\|2\|3） | 5\|5\|7 |  |
| Dyskinesias（1\|2\|3） | 5\|12\|0 |  |
| Dystonia（1\|2\|3） | 4\|10\|3 |  |

Yes=1; No=2; Unknown=3.
