# Supplementary material for: Identification of ferroptosis related biomarkers and immune infiltration in Parkinson’s disease by integrated bioinformatic analysis
Source: BMC Med Genomics. 2023 Mar 14;16:55. doi: 10.1186/s12920-023-01481-3 (PMC10012699; doi:10.1186/s12920-023-01481-3)
Supplement: Supplementary file 2 — Supplementary Material 2 [file 12920_2023_1481_MOESM2_ESM.docx]

Supplemental Table 2 The immune checkpoint genes expressed on T cells.

| Symbol | Gene ID | Gene name |
| --- | --- | --- |
| TNFRSF18 | 8784 | TNF receptor superfamily member 18 |
| TNFRSF4 | 7293 | TNF receptor superfamily member 4 |
| TNFRSF25 | 8718 | TNF receptor superfamily member 25 |
| TNFRSF9 | 3604 | TNF receptor superfamily member 9 |
| CD160 | 11126 | CD160 molecule |
| CD244 | 51744 | CD244 molecule |
| CD28 | 940 | CD28 molecule |
| CTLA-4 | 1493 | Cytotoxic T-lymphocyte associated protein 4 |
| ICOS | 29851 | Inducible T cell co-stimulator |
| CD96 | 10225 | CD96 molecule |
| TIGIT | 201633 | T cell immunoreceptor with Ig and ITIM domains |
| BTLA | 151888 | B and T lymphocyte associated |
| CD200R | 131450 | CD200 receptor 1 |
| MYLK | 4638 | Myosin light chain kinase |
| HAVCR2 | 84868 | Hepatitis A virus cellular receptor 2 |
| CD27 | 939 | CD27 molecule |
| LAG-3 | 3902 | Lymphocyte Activating 3 |
| CD226 | 10666 | CD226 molecule |
| TMIGD2 | 126259 | Transmembrane and immunoglobulin domain containing 2 |
| CD209 | 30835 | CD209 molecule |
| CEACAM1 | 634 | CEA cell adhesion molecule 1 |
| SIRPA | 140885 | Signal regulatory protein alpha |
| ADORA2A | 135 | Adenosine A2a receptor |
| CD40L | 959 | CD40 ligand |
| PDCD1 | 5133 | Programmed cell death 1 |
