# Supplementary figures and images for: Identification of ferroptosis related biomarkers and immune infiltration in Parkinson’s disease by integrated bioinformatic analysis
Source: BMC Med Genomics. 2023 Mar 14;16:55. doi: 10.1186/s12920-023-01481-3 (PMC10012699; doi:10.1186/s12920-023-01481-3)

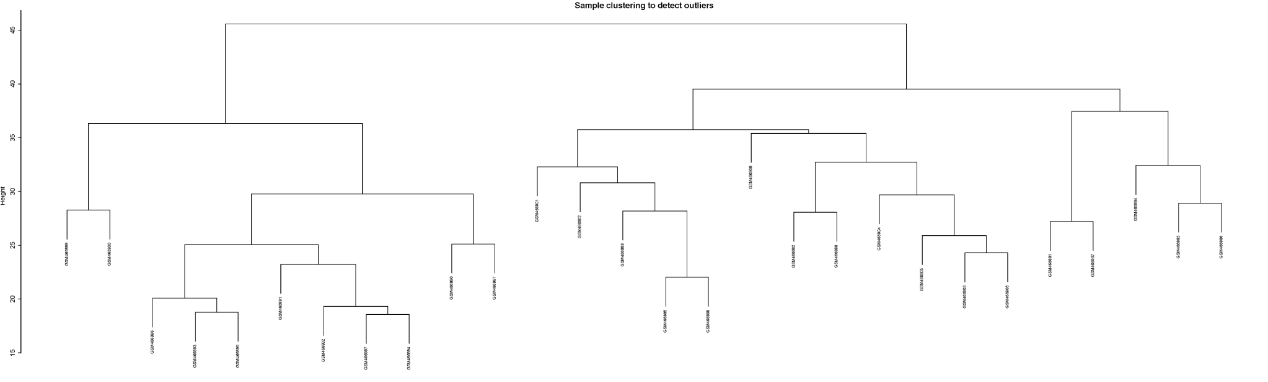


Figure S3

Sample clustering to detect outliers.

Supplement: Supplementary file 3 — Supplementary Material 3 [file 12920_2023_1481_MOESM3_ESM.docx]
