# Supplementary material for: Identification of ferroptosis related biomarkers and immune infiltration in Parkinson’s disease by integrated bioinformatic analysis
Source: BMC Med Genomics. 2023 Mar 14;16:55. doi: 10.1186/s12920-023-01481-3 (PMC10012699; doi:10.1186/s12920-023-01481-3)
Supplement: Supplementary file 4 — Supplementary Material 4 [file 12920_2023_1481_MOESM4_ESM.docx]

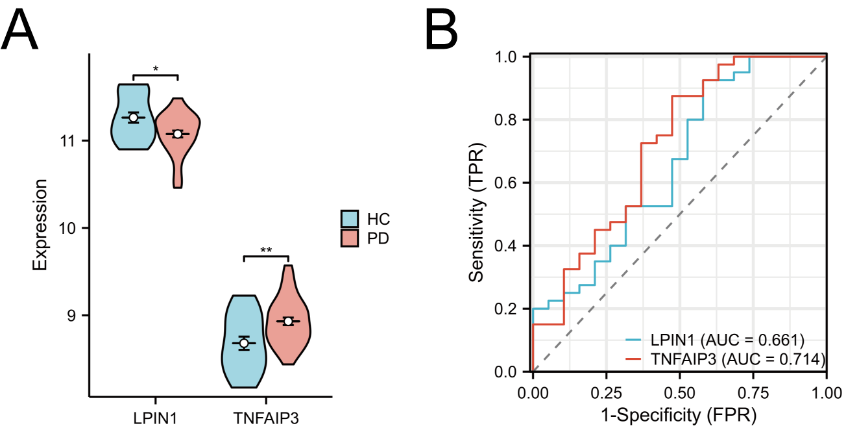


Figure S4

Validation in GSE72267.

**A** The expression levels of LPINI and TNFAIP3 in GSE72267. **B** The ROC curve of two candidate genes. *p<0.05, *p<0.01.
