# Supplementary material for: Identification of ferroptosis related biomarkers and immune infiltration in Parkinson’s disease by integrated bioinformatic analysis
Source: BMC Med Genomics. 2023 Mar 14;16:55. doi: 10.1186/s12920-023-01481-3 (PMC10012699; doi:10.1186/s12920-023-01481-3)
Supplement: Supplementary file 5 — Supplementary Material 5 [file 12920_2023_1481_MOESM5_ESM.docx]

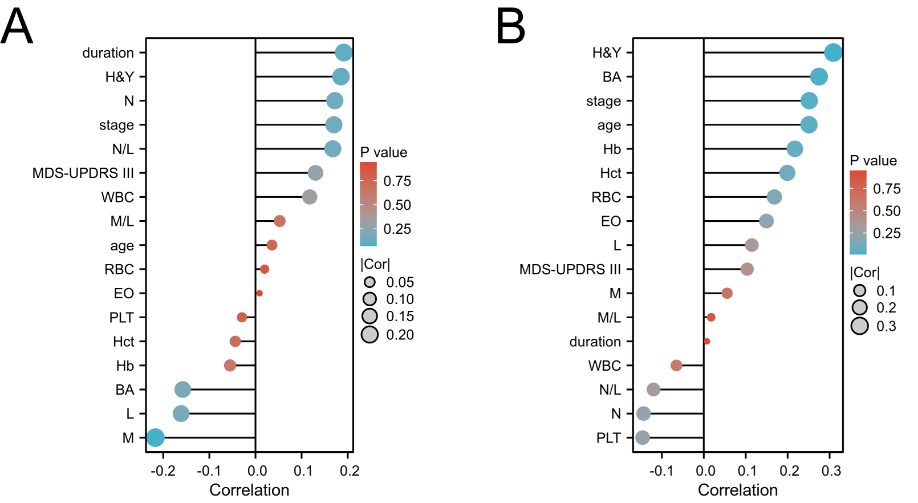


Figure S5

The correlation between LPIN1, TNFAIP3 and clinical characteristics.

**A** The correlation between LPIN1 and clinical characteristics. **B** The correlation between TNFAIP3 and clinical characteristics. BA: basophil, EO: eosinophil.
