# Supplementary material for: Identification of ferroptosis related biomarkers and immune infiltration in Parkinson’s disease by integrated bioinformatic analysis
Source: BMC Med Genomics. 2023 Mar 14;16:55. doi: 10.1186/s12920-023-01481-3 (PMC10012699; doi:10.1186/s12920-023-01481-3)
Supplement: Supplementary file 6 — Supplementary Material 6 [file 12920_2023_1481_MOESM6_ESM.docx]

Supplemental Table 5 Performance of individual indicators or combination models in the detection of PD from healthy controls.

| Indicator | Cutoff | AUC | S.E. | CI 95 | Sens. | Spec. | p |
| --- | --- | --- | --- | --- | --- | --- | --- |
| LPIN1 | 114.503 | 0.754 | 0.048 | 0.659-0.849 | 0.771 | 0.692 | 0.0001 |
| TNFAIP3 | 33.031 | 0.754 | 0.048 | 0.660-0.849 | 0.686 | 0.821 | 0.0001 |
| model | 0.928 | 0.833 | 0.042 | 0.750-0.916 | 0.671 | 0.923 | 0.0001 |

AUC: area under curve; S.E.: standard error; CI 95: 95% confidence interval; Sens.: sensitivity; Spec.: specificity; Cutoff values were calculated according to maximal Youden index.
