# Supplementary material for: Identification of ferroptosis related biomarkers and immune infiltration in Parkinson’s disease by integrated bioinformatic analysis
Source: BMC Med Genomics. 2023 Mar 14;16:55. doi: 10.1186/s12920-023-01481-3 (PMC10012699; doi:10.1186/s12920-023-01481-3)
Supplement: Supplementary file 7 — Supplementary Material 7 [file 12920_2023_1481_MOESM7_ESM.docx]

Supplemental Table 6 Performance of individual indicators or combination models in the detection of early PD patients from healthy controls.

| Indicator | Cutoff | AUC | S.E. | CI 95 | Sens. | Spec. | p |
| --- | --- | --- | --- | --- | --- | --- | --- |
| LPIN1 | 114.167 | 0.817 | 0.051 | 0.717-0.917 | 0.867 | 0.692 | 0.0001 |
| TNFAIP3 | 28.228 | 0.650 | 0.073 | 0.507-0.794 | 0.667 | 0.718 | 0.040 |
| model | -0.678 | 0.831 | 0.049 | 0.734-0.927 | 0.900 | 0.692 | 0.0001 |

AUC: area under curve; S.E.: standard error; CI 95: 95% confidence interval; Sens.: sensitivity; Spec.: specificity; Cutoff values were calculated according to maximal Youden index.
