# Supplementary material for: Identification of ferroptosis related biomarkers and immune infiltration in Parkinson’s disease by integrated bioinformatic analysis
Source: BMC Med Genomics. 2023 Mar 14;16:55. doi: 10.1186/s12920-023-01481-3 (PMC10012699; doi:10.1186/s12920-023-01481-3)
Supplement: Supplementary file 8 — Supplementary Material 8 [file 12920_2023_1481_MOESM8_ESM.docx]

Supplemental Table 7 Performance of individual indicators or combination models in the detection of early from mid-advanced PD patients.

| Indicator | Cutoff | AUC | S.E. | CI 95 | Sens. | Spec. | p |
| --- | --- | --- | --- | --- | --- | --- | --- |
| LPIN1 | 112.724 | 0.599 | 0.068 | 0.465-0.733 | 0.450 | 0.767 | 0.146 |
| TNFAIP3 | 37.528 | 0.647 | 0.070 | 0.510-0.783 | 0.750 | 0.567 | 0.035 |
| model | 0.483 | 0.637 | 0.067 | 0.505-0.768 | 0.450 | 0.800 | 0.041 |

AUC: area under curve; S.E.: standard error; CI 95: 95% confidence interval; Sens.: sensitivity; Spec.: specificity; Cutoff values were calculated according to maximal Youden index.
